# Esophageal Protection and Temperature Monitoring Using the Circa S-Cath™ Temperature Probe during Epicardial Radiofrequency Ablation of the Pulmonary Veins and Posterior Left Atrium

**DOI:** 10.3390/jcm11236939

**Published:** 2022-11-25

**Authors:** Rani Kronenberger, Orlando Parise, Ines Van Loo, Sandro Gelsomino, Ashley Welch, Carlo De Asmundis, Mark La Meir

**Affiliations:** 1 Department of Cardiac Surgery, Universitair Ziekenhuis Brussel, Laarbeeklaan 101, 1090 Brussels, Belgium; 2 Cardiovascular Research Institute Maastricht (CARIM), Maastricht University Medical Centre, Universiteitssingel 50, 6229 ER Maastricht, The Netherlands; 3 Heart Rhythm Management Centre, Postgraduate in Cardiac Electrophysiology and Pacing, Universitair Ziekenhuis Brussel, Laarbeeklaan 101, 1090 Brussels, Belgium

**Keywords:** ablation, atrial fibrillation, atrio-esophageal fistula, circa, luminal esophageal temperature monitoring

## Abstract

Although epicardial bipolar radiofrequency ablation should diminish the risk of esophageal thermal injury in comparison to an endocardial ablation, cases of lethal atrio-esophageal fistula have been reported. To better understand this risk and to reduce the possibility of a thermal injury, we monitored the esophageal temperature with the Circa S-Cath™ temperature probe during and immediately after the ablation while implementing three procedural safety measures. Twenty patients (15 males; 63 ± 10 years) were prospectively enrolled (November 2019–February 2021). All patients underwent an epicardial ablation procedure, including an antral left and right pulmonary vein isolation with bidirectional bipolar clamping, and a roof and inferior line using unidirectional bipolar radiofrequency. Three procedural preventive mitigations were implemented: (1) transesophageal echocardiographic visualization of the atrio-esophageal interface, with probe retraction before the energy delivery; (2) lifting the ablated tissue away from the esophagus during an energy application; and (3) a 30 s cool-off and irrigation period after the energy delivery. The esophageal temperature was recorded using an insulated multisensory intraluminal esophageal temperature probe (Circa S-Cath™). Of the 20 patients enrolled, 7 patients had paroxysmal atrial fibrillation (AF), 8 persistent AF and 5 longstanding persistent AF. The average maximum luminal esophageal temperature observed was 36.2 ± 0.7 °C (34.8–38.2 °C). In our clinical experience, no abrupt increase in the luminal esophageal temperature above the baseline was observed. Since no measurements exceeded the threshold of 39 °C, no prompt interruption of energy delivery was required. Intraluminal esophageal temperature monitoring is feasible and can be helpful in confirming correct catheter position and safe energy application in bipolar epicardial left atrial ablation. Intra-procedural preventive mitigations should be implemented to reduce the risk of esophageal temperature rises.

## 1. Introduction

In patients with symptomatic, drug-refractory atrial fibrillation (AF) whose health and quality of life is impaired, catheter ablation (CA) should be considered as a long-term rhythm control management strategy. The efficacy of different approaches (percutaneous, epicardial, hybrid, point by point and single shot device), together with the potential complications, should be thoroughly discussed with the patient. The 2020 ESC guidelines state that approximately 4–14% of patients undergoing an AF ablation experience complications [1]. These may occur within the first 24 h after the procedure, or even up to 1–2 months after the ablation. Complications may vary from being minor to potentially life-threatening. The authors therefore advise that “patients must be fully informed about the clinical signs and symptoms of rare yet potentially dangerous ablation-related complications that may occur after hospital discharge (e.g., atrio-esophageal fistula, pulmonary vein stenosis)”. Esophageal thermal lesions (ETL) can be diagnosed by esophagoscopy in up to 47% of patients following a radiofrequency CA [2]. Although most thermal injuries resolve without clinical sequelae, lesions may progress to a fistula (<0.5% for CA, non-available for thoracoscopic procedures) with a mortality rate that might exceed 80% in severe cases [3]. The close vicinity of the esophagus to target sites along the pulmonary veins and the posterior wall of the left atrium plays a crucial role in mediating heat transfer. To reduce the risk of ETL, intraluminal esophageal temperature monitoring (IETM) is considered to be one of the preventive measures to guide titration of power and duration of the energy delivery. Although this method is frequently used in percutaneous CA, it has not yet been applied to a thoracoscopic epicardial AF treatment using bipolar RF energy. Preventive mitigations to limit the risk for a heat transfer during endocardial CA have been described, such as the pre- and peri-procedural imaging of the atrio-esophageal anatomy, power limitation when performing an inferior line, the frequent motion of the ablation catheter, the displacement of the esophagus and the intra-luminal esophageal cooling. We sought to find similar, efficacious preventive measures to apply during the epicardial ablation. Rather than first observing a rise in the temperature and then manipulating the catheter or the energy delivery as is often employed in a percutaneous approach [4], we looked for strategies that directly avoided inappropriate luminal temperature rises.

This prospective study was designed to evaluate the effect of three preventive measures on the esophageal temperature using the Circa S-Cath™ multi-sensor IETM probe to detect a potential increase in the temperature that could lead to ETL. Secondly, since saline irrigation of the pericardial space reduces the temperature of the atrio-esophageal space, we studied the potential effect of preventive thermal protection by epicardial irrigation to see if the esophageal temperature could be reduced below the body temperature.

## 2. Materials and Methods

### 2.1. Patient Population

Twenty patients with symptomatic, drug-refractory AF, undergoing a thoracoscopic epicardial RF ablation, were enrolled in this study. The performed lesion set was standardized and consisted of a bipolar ablation of the left and right pulmonary veins (PVs) using a clamping tool (Synergy System^®^; AtriCure, Mason, OH, USA), and a bipolar unidirectional ablation of a roof and inferior line (CoolRail^®^; AtriCure, Mason, OH, USA).

### 2.2. Pre-Procedural Management

A pre-procedural transthoracic echocardiogram, cardiac computed tomography (CT), as well as an electrocardiography and pulmonary function test was performed. A perioperative transesophageal echocardiography (TEE) was done to exclude a possible thrombus in the left atrium.

### 2.3. Esophageal Temperature Monitoring

A Circa S-Cath™ esophageal temperature probe with 12 sensors (Circa Scientific, Inc., Englewood, CO, USA) was used to continuously record the intraluminal esophageal temperature. According to Tschabrunn et al., multi-sensor esophageal probes provide a superior dynamic profile compared to single sensor probes, allowing for the more frequent recognition of temperature increase with an earlier detection time, steeper rising slope, and higher peak temperature [5]. After an endotracheal intubation, the probe was inserted by the anesthesiologist and properly positioned under fluoroscopic guidance into the area at risk of the esophagus. It was advanced straightened (with its stylet) until the temperature sensors spanned the posterior wall of the left atrium from the tracheal carina downwards. Then, the stylet was removed, and the now sinusoidal-shaped probe visualized fluoroscopically to ensure an adequate positioning. The 12 temperature sensors are 2.5 mm in length and separated by approximately 10 mm. Since the probe’s profile is flexible and shapes into an S profile, it has the ability of delivering data from the full length and width of the portion of the esophageal lumen that is exposed to a thermal threat. The advantage of the design is that it probably avoids the need to adjust the probe position during ablation. Once connected to the Circa Temperature Monitoring System (CS-1000 Circa Temperature Monitoring System, Circa Scientific, Inc., Englewood, CO, USA) a continuous maximum temperature is displayed. The thermistor accuracy is ±0.3 °C.

### 2.4. Ablation Procedure

All procedures were performed through a left-sided video-assisted thoracoscopic approach under general anesthesia with selective right lung ventilation. This technique has previously been published by Maesen et al. [6]. In summary, the AF ablation was performed on the beating heart. Before the incision, the absence of a left atrial appendage thrombus was confirmed on the TEE. Three 5 mm ports were introduced into the left hemithorax. The lateral pericardium was opened and bulging of the TEE probe through the posterior pericardium was visualized posterior to the left atrium, to understand the position of the esophagus with regard to the PVs and the inferior line. The TEE was then retracted at 20 cm from the teeth to prevent potential thermal injury of the esophagus by RF or heat transfer during the ablation. The antral ablation of the left PVs with the bipolar RF pulmonary veins clamp (Synergy System^®^; AtriCure, Mason, OH, USA) was done, followed by a roof and inferior line with a unidirectional bipolar RF rail device (CoolRail^®^; AtriCure, Mason, OH, USA), to create an ablation line that connects both superior and inferior PVs. While performing these linear lesions, the ablated cardiac tissue was lifted away from the esophagus during the energy application, followed by a 30 s cool-off period after the energy delivery with the irrigation of the linear catheter, the ablated tissue, and the surrounding tissues by saline at room temperature. Energy delivery was to be discontinued when the maximum intraluminal esophageal temperature on any sensor of the probe rose abruptly or exceeded 39 °C. The posterior box was completed by an antral ablation of the right PVs with the bipolar RF pulmonary veins clamp. During the whole procedure, the LET was measured by the Circa S-Cath^®^. To finalize, we closed the left atrial appendage with a clip (AtriClip^®^; AtriCure, Mason, OH, USA).

### 2.5. Post-Procedural Management

Postoperatively, patients were sent to the intensive care unit. Low-molecular-weight heparin was started 6 h after the procedure, and oral anticoagulation or non-vitamin K antagonist oral anti-coagulants were reinitiated on postoperative day four. A proton pump inhibitor was commenced for four weeks. Oral anticoagulation and antiarrhythmic drugs were continued for at least 3 months. Before the patients’ discharge, a transthoracic echocardiogram was performed to exclude post-operative pericardial effusion.

### 2.6. Follow-Up

Before their discharge, patients were educated extensively on the signs and symptoms of an esophageal injury, as published by our group to ensure a postoperative clinical vigilance [7]. After their discharge, the patients were seen in the outpatient arrhythmia clinic at 1, 3, 6, 12 and 24 months for a clinical evaluation and to assess any possible procedure-related complications.

### 2.7. Approval

Participation in the study was voluntarily and all participants provided written informed consent prior to their enrollment. The study was approved by the Ethical Medical committee of the University Hospital of Brussels.

## 3. Results

### 3.1. Patient Characteristics

Twenty patients (15 males; 63 ± 10 years) were prospectively enrolled in the study. There were seven patients with paroxysmal AF, eight with persistent AF and five with longstanding persistent (LSP) AF. Sixteen patients underwent ≥1 previous endocardial CA. All patients had failed ≥1 Class I or III antiarrhythmic drugs (AAD). The mean BMI was 27.4 ± 4.9 (18.7–36.2). The average left ventricular ejection fraction (LVEF) was 56 ± 10% (30–60%) and the average CHA2DS2-VASc score was 2 ± 1.4 (0–4). The comorbidities included hypertension (13), a previous transient ischemic attack (TIA), stroke or thromboembolism (TE) (4), diabetes (2) and vascular disease (2). All the patient baseline clinical characteristics are listed in Table 1.

### 3.2. Esophageal Temperature Monitoring

**Figure 1:**
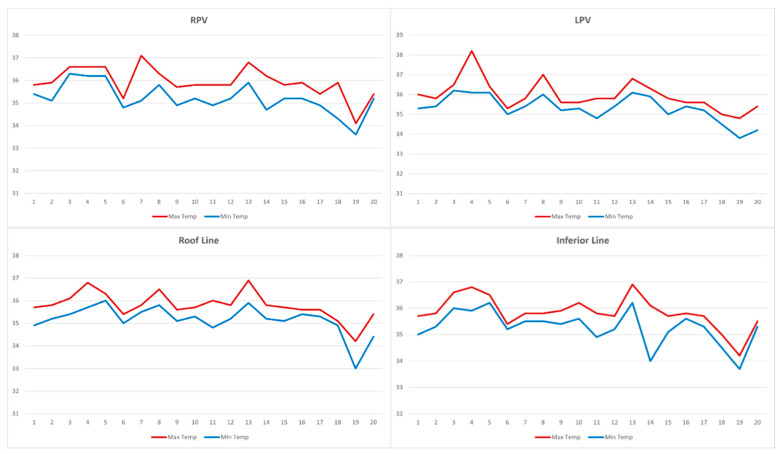
Maximal and minimal esophageal temperatures measured during the ablation procedure. Abbreviations: RPV: right pulmonary vein; LPV: left pulmonary vein. The x-axis is the patient number; the y-axis is luminal esophageal temperature (°C).

In our clinical experience, no abrupt increase in the LET above the baseline was observed. Using the Circa S-Cath™, the average maximum LET observed in all the patients was 36.2 ± 0.7 °C (34.8–38.2 °C). The maximum measured temperature was 38.2 °C (<39 °C) during the ablation of the LPV, thus, no cessation of energy delivery was required. The average minimum LET observed in all the patients was 35 ± 0.7 °C (33.0–36.0 °C). Table 2 and Figure 1 summarize the maximal and minimal temperature of all the study patients.

### 3.3. Procedural Complications

No peri-procedural complications occurred in the study population. One patient developed postoperative pericarditis, successfully treated with colchicine and non-steroidal anti-inflammatory drugs. One patient complained of epigastric pain, for which an esophagoscopy was performed in the early postoperative stage to exclude an esophageal thermal injury. Erosive antritis was diagnosed. The microscopic work-up revealed a non-active, mild chronic (chemical) gastritis. No lesions related to the ablation procedure were seen. Furthermore, no major complications, including death, thromboembolic events, pacemaker insertion, pericardial effusion or tamponade occurred in the postoperative period.

### 3.4. Follow-Up

The arrhythmia results are presented in accordance with the 2020 ESC Guidelines for AF [1]. The mean follow-up was 569 ± 328 days (42–960). No late procedural complications occurred. The recurrence of arrhythmia was seen in three patients: one patient with AF, one paroxysmal atrial tachycardia and one mitral isthmus dependent flutter. All three recurrences were successfully treated with a redo catheter ablation.

## 4. Discussion

The primary indication for stand-alone thoracoscopic epicardial or hybrid ablation is symptomatic AF, refractory or intolerant to at least one Class I or Class III AAD. For paroxysmal AF, most patients will have had at least one unsuccessful CA. For persistent and LSP AF, the primary referral of a patient is often associated with a presumed high risk for percutaneous failure. In order to improve the sinus rhythm outcome in this difficult to treat population, most surgeons will create, apart from pulmonary vein isolation (PVI), a linear lesion between the superior and inferior PVs to isolate the posterior wall of the LA. This so-called box lesion could increase the risk of esophageal thermal lesions. The use of temperature probes during RF ablation on the LA posterior wall has the potential to reduce the risk of ETL by restricting the energy delivery if the maximum LET rises abruptly or exceeds a predefined limit (often 39 °C). A review of the published literature on atrio-esophageal fistula after a surgical ablation learns that none of these patients received esophageal temperature measurements during the procedure. There is only one surgical AF procedure where esophageal temperature recording is advised, namely during the epicardial Episense^®^ (AtriCure, Mason, OH, USA) ablation of the posterior LA wall. A major difference between the surgical and percutaneous RF catheters is the use of bipolar ablation technology. Therefore, energy is not directed towards a ground pad on the back of the patient, potentially driving it through the esophagus, TEE or the luminal temperature probe. Bipolar ablation tools focus their energy between the two conduction electrodes of the ablation device, which theoretically prevents an energy dispersion, thus, the formation of ETL. In epicardial ablation, the RF is directed towards the atrial tissue from outside to inside. Although an epicardial ablation using bipolar RF energy is generally considered safe and should avoid an esophageal thermal injury, it has been associated with the formation of an AEF. Another important difference between percutaneous catheters and surgical ablation tools is that in the latter, the titration of power is not possible. The surgical generators do not allow for a presetting of the power or impedance, as this is always automatically controlled. Only the duration of an energy application can therefore be decided upon.

The close anatomic relation between the esophagus and the posterior left atrium plays a pivotal role in acute esophageal thermal injury during an endocardial and epicardial ablation. Furthermore, the esophagus is the only gastrointestinal organ that lacks an outer serosal layer. Therefore, to avoid a potential ETL formation by energy or heat transfer, understanding the anatomy of the atrio-esophageal interface is crucial. Sánchez-Quintana et al. studied this anatomy and histology in cadavers and human heart specimens [8]. The esophageal route varies individually due to its displacement by the aortic arch, resulting in two common routes: adjacent to the left or right inferior PVs. Since the esophagus follows a route posterior to the LA through the supero-posterior mediastinum towards the esophageal hiatus of the diaphragm, both structures share a mean contact length of 42 ± 7 mm (range 30–53 mm). Moreover, the atrio-esophageal interface is <5 mm in 40% of cases. The atrial wall is thinnest at the superior level where the mean atrio-esophageal distance is 2.3 ± 1.2 mm (range, 1 to 8.2 mm). The posterior LA wall is thickest inferiorly, with an average thickness of 6.5 ± 2.5 mm. Several studies have elucidated that the posterior LA wall near the left inferior PV orifice is the most common site of an AEF. When an epicardial roof line is made, the superior PVs are connected by an ablation line just below the Bachmann bundle. At this level, the posterior portion of the LA roof functions as a spatial buffer between the epicardial ablation tool and the esophagus. As a result, the left atrial roof line is never in a close vicinity to the esophagus. Contrarily, when an inferior line ablation is applied by connecting the inferior PVs, the line is positioned posteriorly. In contrast to the roof line, there is no remaining spatial buffer, thus carrying an increased risk for ETL. Avoiding high-risk zones in close proximity to the esophagus while deciding on the location of the ablation lines around the PV ostia and LA is important to prevent ETL.

One of the advantages of an endoscopic approach is that it guarantees the visually controlled contact of the ablation catheter with the left atrium and the PVs. All epicardial surgical RF ablation devices have been designed to only deliver energy on the area in contact with the atrial tissue. The backside of the catheter facing the posterior pericardium is isolated and cannot transfer RF energy to the surrounding tissues. Therefore, when correctly positioned away from the posterior epicardial space, there should be no risk of ETL. This seems true for bipolar clamping devices, but not for the bipolar and monopolar linear devices. When these ablation catheters are removed immediately after the energy delivery, the high atrial tissue temperature has the capacity to transmit heat through the convection to the adjacent structures for approximately 30 s. Thus, if the heated atrial tissue touches the pericardium overlying the esophagus, it acts as a heat sink, potentially causing a thermal injury. To avoid this, Kronenberger et al. proposed three mitigation strategies to minimize this risk [7]. A direct videoscopic inspection and pericardial access allows for the confirmation of the position of the esophagus (bulging of the TEE probe through the posterior pericardium) with regard to the left atrial posterior wall by advancing and retracting the TEE probe prior to an ablation. Manually pushing the stiff shaft of the ablation tool upwards will create a space between the ablation catheter and the posterior pericardium during and immediately after the energy delivery. Furthermore, to avoid a thermal spread from the ablated tissue, a 30 s cool-off period after the energy delivery with the irrigation of the linear catheter, the ablated tissue and the surrounding tissues is performed. The active cooling of the area with room temperature saline should be helpful to avoid an esophageal thermal injury since the esophagus is cooled below the body temperature. This strategy is easily applied and is based upon the principle of preventive thermal protection (local cooling) of the atrio-esophageal interface with a mechanical deviation of the ablated tissue rather than the deviation and intraluminal cooling of the esophagus.

Thermal protection during an endocardial RF ablation has been studied by Yeung et al. in the IMPACT Study [9]. The authors performed a 1:1 randomization, comparing a control group of the standard practice utilizing a single-point temperature probe and a protected group where a device cooled the luminal temperature at 4 °C during an RF ablation. Mucosal thermal injury was significantly more common in the control group than in those receiving esophageal protection (12/60 vs. 2/60; *p* = 0.008). The authors concluded that thermal protection of the esophagus significantly reduces the occurrence of an ablation-related thermal injury compared with standard care. Although often proposed and studied, there is still no consensus on the add-on value of esophageal probes for LET monitoring during a CA. In a paper by Halbfass et al., evaluating the effects of the Circa S-Cath™, the authors concluded that the use of esophageal temperature probes with insulated thermocouples seems to be feasible in patients undergoing an RF ablation [10]. The incidence of post-procedural endoscopically detected esophageal lesions (EDEL) when using a cut-off of 39 °C was comparable to the incidence of EDEL without using a temperature probe [10]. However, LET monitoring is not without controversy. Singh et al. reported that the use of LET temperature probes may be detrimental by serving as a heat sink via a thermal conduction [11]. Therefore, although LET monitoring in epicardial ablation is feasible and could be a helpful tool for warning the surgeon in case of inappropriate esophageal heating, we emphasize the need for further studies to confirm the safety of using LET probes during ablation.

Although most mitigation strategies seem easy to apply, an epicardial AF ablation needs surgical endoscopic skills that require proper training. While often discussed, there is no specific mandatory program required for a surgeon to perform a minimally invasive surgical AF ablation. If we want to achieve the best possible outcome for patients, by obtaining long-lasting sinus rhythm and reducing the complication rates, it is necessary to teach surgeons to treat stand-alone AF patients using catheter and left atrial appendage closure technologies. The AATS expert consensus guidelines highly recommends surgeons who are new to surgical AF to be proctored by an experienced surgeon for three to five cases before performing a surgical ablation alone [12]. During this training, LET could be a helpful tool that could warn the surgeon of inappropriate esophageal heating, thereby ensuring that the mitigation strategies have been correctly applied. With experience, the surgeon will be able to correctly visualize and safely position the ablation catheters so that the three mitigation strategies by themselves should be sufficient to avoid any risk of an esophageal thermal injury.

## 5. Conclusions

If three simple preventive measures are correctly applied during thoracoscopic epicardial bipolar RF ablation, there should be no risk for an esophageal thermal injury, as confirmed by the temperature measurements using the Circa S-Cath™ multi-sensor IET probe. The combination of (I) the inspection of the atrio-esophageal interface during ablation and TEE probe retraction, (II) displacement of the ablated tissue and (III) thermal protection of the esophagus by infusing room-temperature saline into the posterior pericardial space, minimizes the risk of heat dissipation. Since specific surgical endoscopic skills are required to perform this procedure safely, we would advise a standardized use of CircaS-Cath™ at least during the training period. In conclusion, prevention combined with the cooling protection and intraluminal esophageal temperature monitoring yields promising results in avoiding ETL during an epicardial RF ablation.

## 6. Limitations

Due to the limited number of patients and a small data set, our study does not allow for generalized conclusions. Further on, no systematic postoperative esophagoscopy was performed. However, in a previous paper by our research group [7], the postoperative esophageal findings were studied after using the same perioperative mitigation strategies. No esophageal thermal lesions were observed. Furthermore, to better value the benefit of esophageal temperature monitoring, a study including a control group without mitigation strategies, preferably randomized, would have to be performed. However, having seen the potentially detrimental risk for ETL, it was not deemed ethical to compare the esophageal temperatures with and without preventive strategies. Larger studies are required to better understand and estimate the risk of an esophageal injury in an epicardial RF ablation.

## Figures and Tables

**Table 1:** Baseline clinical characteristics.

Baseline Characteristics	*n =* 20
Age, y	63 ± 10 years (35–74)
Gender (Male)	15 pts
Paroxysmal AF	7 pts
Persistent AF	8 pts
Long-standing persistent AF	5 pts
BMI, kg/m^2^	27.4 ± 4.9 (18.7–36.2)
LVEF, %	56 ± 10 (30–60)
CHA_2_DS_2_-VASc Score	2 ± 1.4 (0–4)
Female gender	5 pts
Age ≥ 65 & ≤ 74	9 pts
Age > 75	1 pts
CHF	0 pts
HTN	13 pts
DM	2 pts
CVA/TIA/TE	4 pts
Vascular disease	2 pts
Previous CA	16 pts

Categorical variables are expressed as absolute. Continuous variables are expressed as mean + SD (range). Abbreviations: AF = atrial fibrillation; BMI = body mass index; CA = catheter ablation; CVA = cerebral vascular accident; DM = diabetes mellitus; HTN = hypertension; TIA = transient ischemic attack; TE = thromboembolism; LVEF = left ventricular ejection fraction; CHF= congestive heart failure.

**Table 2:** Maximal and minimal esophageal temperatures measured.

Patient	Patient Max/Min Temperature (°C)	RPV Max/Min (°C)	LPV Max/Min (°C)	Roof Line Max/Min (°C)	Inferior Line Max/Min (°C)
1	36.0/34.9	35.8/35.4	36.0/35.3	35.7/34.9	35.7/35.0
2	35.9/35.4	35.9/35.1	35.8/35.4	35.8/35.2	35.8/35.3
3	36.6/35.4	36.6/36.3	36.5/36.2	36.1/35.4	36.6/36.0
4	38.2/35.7	36.6/36.2	38.2/36.1	36.8/35.7	36.8/35.9
5	36.6/36.0	36.6/36.2	36.4/36.1	36.3/36.0	36.5/36.2
6	35.4/34.8	35.2/34.8	35.3/35.0	35.4/35.0	35.2/35.4
7	37.1/35.1	37.1/35.1	35.8/35.4	35.8/35.5	35.5/35.8
8	37.0/35.5	36.3/35.8	37.0/36.0	36.5/35.8	35.8/35.5
9	35.9/34.9	35.7/34.9	35.6/35.2	35.6/35.1	35.9/35.4
10	36.2/35.2	35.8/35.2	35.6/35.3	35.7/35.3	36.2/35.6
11	36.0/34.8	35.8/34.9	35.8/34.8	36.0/34.8	35.8/34.9
12	35.8/35.2	35.8/35.2	35.8/35.4	35.8/35.2	35.7/35.2
13	36.9/35.9	36.8/35.9	36.8/36.1	36.9/35.9	36.9/36.2
14	36.3/34.0	36.2/34.7	36.3/35.9	35.8/35.2	36.1/34.0
15	35.8/35.0	35.8/35.2	35.8/35.0	35.7/35.1	35.7/35.1
16	35.9/35.2	35.9/35.2	35.6/35.4	35.6/35.4	35.8/35.6
17	35.7/34.9	35.4/34.9	35.6/35.2	35.6/35.3	35.7/35.3
18	35.9/34.3	35.9/34.3	35.0/34.5	35.1/34.9	35.0/34.5
19	34.8/33.0	34.1/33.6	34.8/33.8	34.2/33.0	34.2/33.7
20	35.5/34.2	35.4/35.2	35.4/34.2	35.4/34.4	35.5/35.3

Abbreviations: RPV: right pulmonary vein; LPV: left pulmonary vein.

## Data Availability

The data presented in this study are available on request from the corresponding author. The data are not publicly available due to GDPR privacy restrictions.

## Abbreviations

AAD	Antiarrhythmic drugs
AEF	Atrio-esophageal fistula
AF	Atrial fibrillation
BMI	Body mass index
CA	Catheter ablation
CHF	Congestive heart failure
CVA	Cerebrovascular accident
DM	Diabetes mellitus
ETL	Esophageal thermal lesions
HTN	Hypertension
IETM	Intraluminal esophageal temperature monitoring
LA	Left atrium
LAA	Left atrial appendage
LPV	Left pulmonary Vein
LVEF	Left ventricular ejection fraction
PV	Pulmonary vein
PVI	Pulmonary vein isolation
RPV	Right pulmonary Vein
TE	Thromboembolism
TEE	Transesophageal echocardiogram
TIA	Transient ischemic attack
